# Intraventricular ganglioglioma: a rare case report

**DOI:** 10.1093/jscr/rjad235

**Published:** 2023-04-28

**Authors:** Constantinos Thoma, Gráinne McKenna

**Affiliations:** Barts & The London School of Medicine & Dentistry, London E1 2AD, UK; Neurosurgical Department, Royal London Hospital, London E1 1FR, UK

**Keywords:** neurosurgery, ganglioglioma, intraventricular

## Abstract

We report a case of an intraventricular ganglioglioma in a 23-year-old male. The patient presented with a 3-day history of headache and vomiting. Preoperative brain imaging revealed a calcified lesion within the trigone of the right lateral ventricle, with irregular enhancement, causing entrapment of the temporal horn of the lateral ventricle. At surgery, the lesion was haemorrhagic, easily friable and exhibited evidence of a previous recent haemorrhage. Histological and immunohistochemical studies showed a ganglioglioma with World Health Organisation Grade 1 characteristics. Gangliogliomas of the central nervous system are uncommon, and rarely occur in the lateral ventricle.

## INTRODUCTION

Gangliogliomas are low-grade tumours that contain both neuronal and glial elements [1]. They account for approximately 1% of all intracranial neoplasms [2, 3]. These tumours usually occur in children and young adults under the age of 30 [4]. They are most commonly located in the temporal lobe; however, they have been reported to occur throughout the CNS [5]. These tumours rarely occur within the lateral ventricle. To our knowledge, there have been only 27 reported cases, including ours, of gangliogliomas in this location [6, 7].

## CASE PRESENTATION

### History and examination

A 23-year-old male presented with a 3-day history of left sided headache which was worse on lying down, associated with vomiting, photophobia and left eye pain. His past medical history included surgery at birth for dorsal dysraphism (horizontal lumbar scar noted); however, no further details on the type of dysraphism were available. There was no significant family history. He was a non-smoker who took no regular medications and had no known allergies.

On examination, the patient had a GCS of 15, with pupils equal and reactive to light and accommodation. Ocular pain was noted upon moving the eyes side to side. Cranial nerves II-XII were intact, with normal tone and 5/5 power for all muscle groups in both upper and lower limbs. There was no evidence of dysmetria, dysdiadochokinesis or nystagmus. Visual fields were intact; however, papilloedema was noted bilaterally on fundoscopy.

### Imaging

**Figure 1:**
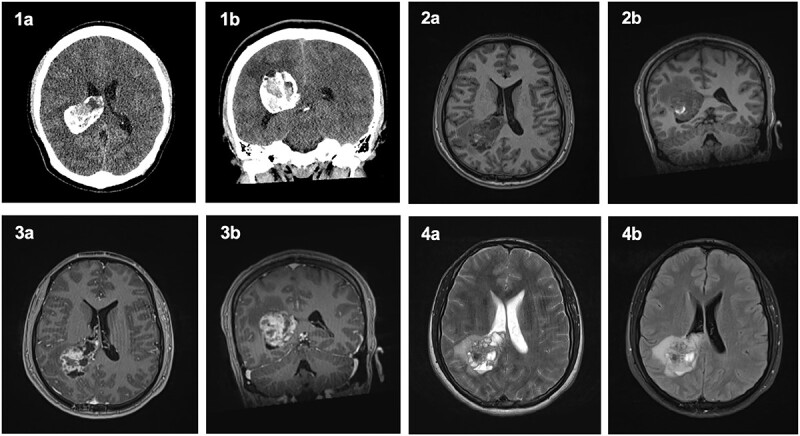
Computed Tomography and Magnetic Resonance Imaging showing a mass within the trigone of the right lateral ventricle. On CT, axial (**1a**) and coronal (**1b**) sections revealed a calcified mass with a central cystic component. Pre-contrast T1-weighted imaging showed an isointense mass with a central ring of enhancement on axial (**2a**) and coronal (**2b**) slices. Post-contrast T1-weighted imaging with irregular enhancement of the lesion on axial (**3a**) and coronal (**3b**) slices. Axial T2-weighted imaging (**4a**) and FLAIR (**4b**) exhibited an isointense lesion with surrounding oedema.

A non-contrast computed tomography (CT) head revealed a 4 × 5 × 5.3 cm^3^ lesion within the trigone of the right lateral ventricle (Fig. 1). The lesion was largely calcified and had a cystic component. Associated cerebral oedema was noted in the periventricular deep white matter of the right parietal and temporal lobes. There was no evidence of midline shift, and the basal cisterns remained patent. A magnetic resonance imaging (MRI) scan showed a lesion in the trigone of the right lateral ventricle, which was mostly isointense on T1-weighted imaging, with a focus of hyperintensity in the centre of the lesion. On T2- weighted imaging, the lesion was isointense. Upon administration of gadolinium, there was irregular enhancement of the lesion, with scattered, non-enhancing regions (Fig. 1).

### Management

**Figure 2:**
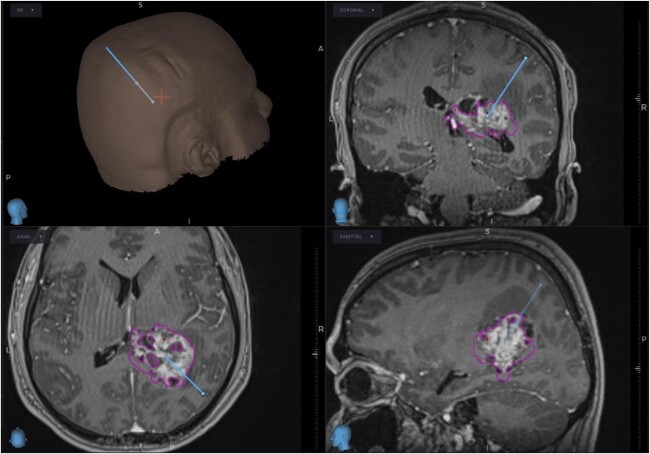
Planned trajectory for excision of the tumour, as seen on the neuronavigation system.

**Figure 3:**
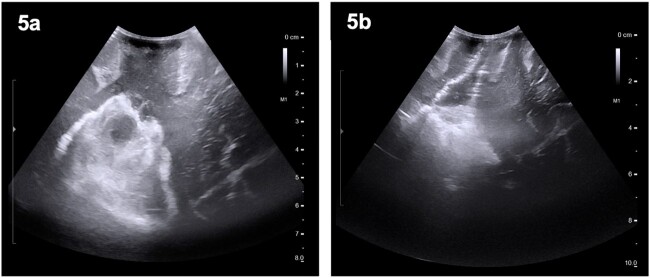
Preoperative (**5a**) ultrasound images depicting a heterogenous mass with hyperechoic and hypoechoic regions. Post-operatively (**5b**), a hyperechoic region is noted; however, no distinct lesion is identified.

**Figure 4:**
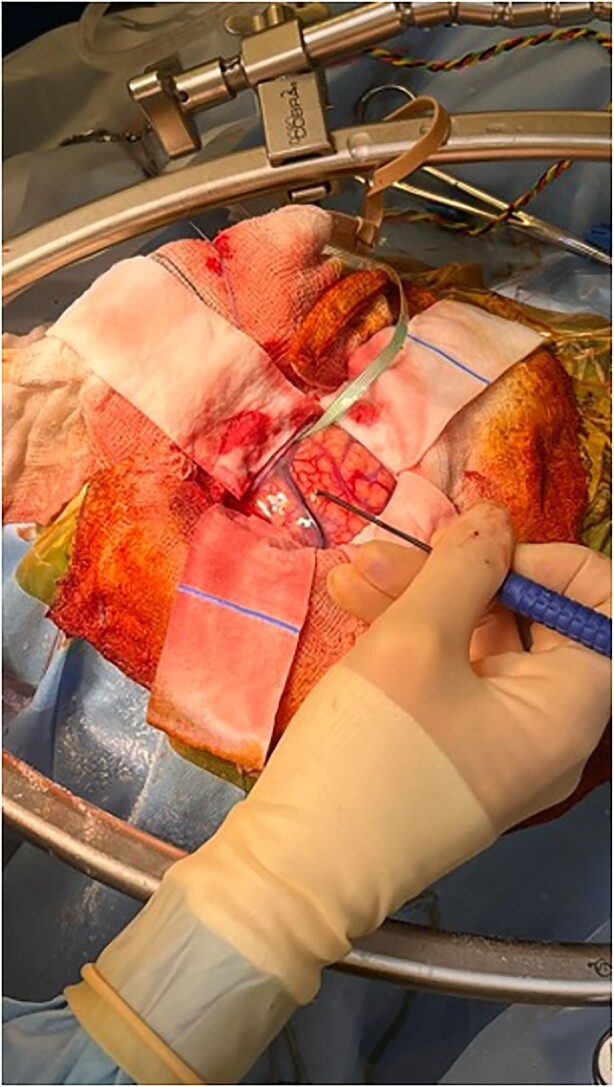
Intraoperative image depicting the use of MEPs to identify the precentral gyrus, prior to insertion of the VBAS. In this image, the anterior aspect of the head is to the right, whereas the posterior aspect is to the left.

The patient was started on oral dexamethasone, resulting in the improvement of his headaches. The differential diagnosis based on imaging findings included an atypical meningioma, central neurocytoma, choroid plexus papilloma or carcinoma, ependymoma or an oligodendroglioma. He underwent a right parietal craniotomy with gross total resection of the lesion. Intraoperatively, use of the Vycor™ ViewSite Brain Access System (VBAS) was utilized, which allowed for optimized surgical site access, and reduced the risk of brain retractor injury to surrounding structures, namely the precentral gyrus. This was combined with intraoperative ultrasound (Fig. 3), which provided real time imaging and guided the extent of the resection. In addition, neuromonitoring and neuronavigation (Fig. 2) were incorporated to further reduce the risk of damage to surrounding structures. As part of the neuromonitoring, motor evoked potentials (MEPs) were performed to identify the precentral gyrus prior to insertion of the VBAS (Fig. 4), ensuring that any resection occurred posterior to the motor strip. During surgery, it was noted that the lesion was haemorrhagic and easily friable, with evidence of a previous recent haemorrhage.

### Histopathology

A specimen was sent for intraoperative smear, and a preliminary diagnosis of a meningioma was made. This was due to the presence of clusters of neoplastic cells with frequent presence of psammoma bodies. However, further analysis of the specimen revealed the presence of both neuronal and glial cell populations. The neuronal cells were large, dysplastic and binucleate, and their nature was confirmed by immunoreactivity to synaptophysin. The glial cell population was characterised by small, elongated nuclei and drawn-out processes. Immunostaining for glial fibrillary acidic protein (GFAP) highlighted the glial component of the specimen. Tumour cell nuclei showed retained expression of ATRX and H3K27me3 IHC. Immunostaining for mutant IDH1 and histone H3 K27M was negative. Given these findings, a diagnosis of a ganglioglioma was made, corresponding to CNS WHO Grade 1. Methylation profiling confirmed the diagnosis of a ganglioglioma and found that the O6-methylguanine DNA methyltransferase (MGMT) promoter was unmethylated. Genetic sequencing identified a BRAF p.V600E mutation.

### Post-operative course

**Figure 5:**
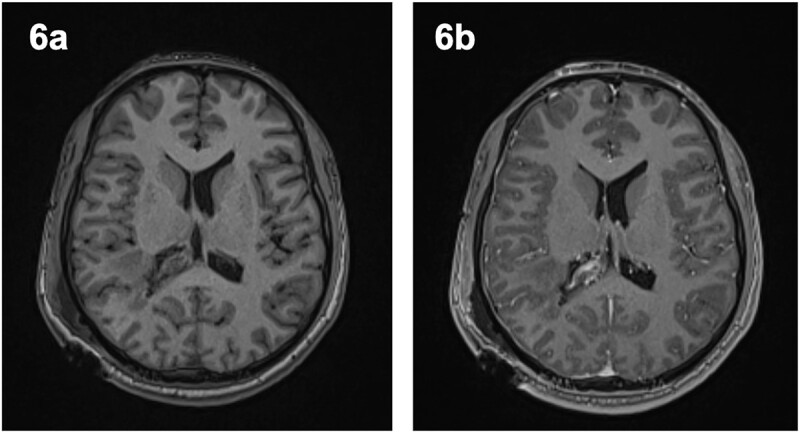
Post-operative magnetic resonance imaging revealed evidence of intraventricular enhancement, with no distinct lesion identified on axial T1-weighted imaging pre- (**6a**) and post-contrast (**6b**).

The patient remained neurologically intact post-operatively and was discharged 5 days after surgery. A post-operative MRI revealed some intraventricular enhancement but no clear residual tumour (Fig. 5), likely indicating a complete resection of the lesion. He was seen in clinic 4 days and again 3 months after discharge and was noted to be recovering well.

## DISCUSSION

Gangliogliomas account for approximately 1% of all intracranial neoplasms [2, 3]. Typically, gangliogliomas are low-grade, calcified tumours with cystic components. They are defined pathologically by the presence of neuronal and glial elements [1, 5]. The neuronal element is composed of ganglion cells, whereas the glial element may resemble oligodendroglioma, fibrillary astrocytoma or pilocytic astrocytoma. Usually, the glial element is benign and corresponds to World Health Organisation Grade 1 [5]. These tumours have a favourable prognosis, with one series noting a 93% 5-year survival rate [8], and another demonstrating that the 2-, 5- and 10-year overall survival for patients with low-grade ganglioglioma was >80% (100, 88 and 84%, respectively) [11].

Typically, gangliogliomas present in children and young adults with focal epilepsy. They are frequently in the supratentorial compartment, most commonly in the temporal lobe [7]. However, they have also been reported to occur in the frontal, parietal and occipital lobes, as well as the brainstem, cerebellum, spinal cord, optic nerves and pineal gland. Gangliogliomas of the lateral ventricles are rare, with only 27 cases described in the literature, including ours [2, 6, 7].

Radiological findings of gangliogliomas are non-specific and exhibit significant heterogenicity. On CT, they are usually hypodense lesions with variable enhancement and calcification [9]. It has been reported that the appearance of these tumours on MRI varies widely, with 40–70% appearing isointense and 20–40% hypointense on T1-weighted imaging. Simultaneously, on T2-weighted imaging, 20–30% of gangliogliomas appear isointense while 70–90% appear hyperintense [6, 7]. Given this, a conclusive diagnosis of ganglioglioma cannot be made based on imaging alone.

The preferred treatment for gangliogliomas is surgical resection. Gross total resection is a positive prognostic factor; however, the extent of resection is dependent on tumour location and intraoperative circumstances. In patients who are ineligible for surgery due to tumour location, gamma knife radiosurgery can be used as an alternative [3, 10]. However, the role of chemotherapy and radiotherapy in the treatment of ganglioglioma remains unclear; thus, gross total resection is considered the gold-standard [12].
